# Late diagnosis of Lassa fever outbreak in endemic areas lead to high mortality, Kenema District, Sierra Leone, February - March 2019

**DOI:** 10.11604/pamj.2022.42.256.35838

**Published:** 2022-08-08

**Authors:** Umaru Sesay, Leonard Hakizimana, Adel Hussein Elduma, Alden Henderson, Gebrekrstos Negash Gebru

**Affiliations:** 1 Field Epidemiology Training Program, Freetown, Sierra Leone,; 2 Centers for Disease Control and Prevention (CDC), Georgia, United States

**Keywords:** Lassa fever, Kenema, Sierra Leone, endemicity

## Abstract

**Introduction:**

the Kenema District Surveillance team in Sierra Leone received notifications of patients with suspected Lassa fever on February 20^th^ and March 2^nd^, 2019. On that day, an investigation started to confirm the diagnosis and search for additional cases.

**Methods:**

we used the Lassa fever surveillance case definition and collected demographic and exposure information from suspected cases through interviews and clinical records. Blood samples were collected from the cases to confirm the diagnosis. Active case finding was conducted in the community and health facility.

**Results:**

on February 10, 2019, an eight-year-old male developed a fever (>39.5°C) and a sore throat. On February 18, 2019, he was admitted to a hospital and treated for malaria and pneumonia. On February 20, 2019, Lassa fever was suspected because the patient was bleeding from orifices and testing. On February 15, a 5-year-old female developed fever and headache and was treated with anti-malarial drugs. On February 26^th^ the high fever re-emerged with severe bleeding from the orifices. She was admitted and treated with antibiotics, confirmed for Lassa fever, and died on March 2, 2019.

**Conclusion:**

the two children had Lassa fever, and no additional cases were identified. We sensitized clinicians on suspicion of Lassa fever to improve early detection and treatment.

## Introduction

Lassa fever is a viral hemorrhagic disease endemic in Sierra Leone, Liberia, Guinea, and Nigeria. *Mastomys natalensis*, the natal multimammate rat, is the main reservoir of Lassa fever [1]. Humans are infected by the Lassa fever virus through exposure to food or utensils contaminated by rodent droppings [2]. Lassa fever can be prevented by avoiding contact with *mastomys natalensis* rodents by putting food in rodent-proof containers or by keeping the home clean [3]. Lassa fever incubation period is between two and 21 days, and symptoms begin with flu-like illness, cough, and fever. These symptoms may progress to abdominal pain, vomiting, diarrhea, and in severe stages including bleeding, edema, and hearing loss [1]. About 80% of Lassa fever cases are asymptomatic and a death rate of 1% [4]. Diagnostic tests include the Enzyme-Linked Immunosorbent Assay which detects IgM and IgG antibodies as well as the Lassa antigen, and real-time polymerase chain reaction (RT-PCR) test which detects Lassa virus ribonucleic acid (RNA) [5]. Lassa fever is endemic in West Africa and accounts for an estimated 300,000 new cases and 5000 deaths every year [6]. In the Eastern province of Sierra Leone, 63 suspected cases of Lassa fever were identified in patients admitted to two hospitals over 2 years and health care workers (HCW) were at an increased risk of infection [7]. In Sierra Leone, Lassa fever is a disease under surveillance and reportable in the weekly integrated disease surveillance and response system (IDSR).

The magnitude of Lassa fever mortality and morbidity is difficult to estimate in Sierra Leone due to challenges in timely detection and diagnosis. Lassa fever is endemic in Kenema District, but the burden of Lassa fever is not known because the disease is often diagnosed as febrile illnesses [8] or illnesses such as viral hepatitis, leptospirosis, rheumatic fever, typhus, and mononucleosis which have similar symptoms. A unique diagnostic feature of Lassa fever is the appearance of patches of white or yellowish exudate and occasionally the appearance of small vesicles or shallow ulcers on the tonsils and pharynx of the patients [9]. In 1976, the Government of Sierra Leone, through the Ministry of Health and Sanitation (MoHS) and support from the United States Centre for Disease Prevention and Control (US CDC) constructed a Lassa fever treatment ward with diagnostic facilities in Kenema District to promptly detect and treat people. Despite this support, outbreaks of Lassa fever continue [10, 11]. On 20^th^ February and 2^nd^ March 2019, the district surveillance unit received alerts about patients with suspected Lassa fever from the hospital staff of Panguma Hospital and the Kenema General Hospital pediatric ward. The surveillance team comprised of field epidemiology training program trainees and members from the Kenema District rapid response team investigated within 24 hours of notification to confirm the diagnosis, identify sources of Lassa fever infection, search for additional cases, and provide appropriate control measures.

## Methods

**Study setting:** these two suspected Lassa fever case patients live in Kenema District, one of the 16 districts in Eastern Province, Sierra Leone. The district has a population of 680,000 and 132 health facilities. The main referral hospital, Kenema Government Hospital (KGH), has the capacity to perform enzyme-linked immunosorbent assay (ELISA) and reverse transcriptase-polymerase chain reaction (RT-PCR) tests on human and animal samples for Lassa fever and other hemorrhagic fever viruses. Kenema Government Hospital has an isolation ward for patients with Lassa fever [12]. The district is a known endemic zone for Lassa fever and it accounts for the world´s highest Lassa fever infection rate [13].

**Case definition:** a working case definition was adapted, from the Lassa fever surveillance case definition from the Sierra Leone MoHS, to investigate these two suspected cases. A suspected case was any person residing in the Kenema District with a fever above 38°C and not responding to appropriate anti-malaria and antibiotic treatment within 72 hours with no localized infection from January 01 to March 31, 2019. A probable case was any suspected case with epidemiological links to a confirmed case or any case in which clinicians suspect Lassa fever. A confirmed case was any person with a positive ELISA or RT-PCR test for Lassa fever and with clinical symptoms of a suspected case.

**Data collection:** a semi-structured questionnaire, with closed and open-ended questions, was adapted from the viral hemorrhagic fever case investigation form of the Sierra Leone MoHS and used for data collection. Demographic and clinical data including exposure history, age, sex, occupation, date of onset, and travel history of 21 days before symptoms onset, were collected from case patients through interview and record review. Active search for cases was conducted at the communities and healthcare facilities to identify additional cases and to determine the scope and magnitude of the infection.

**Laboratory diagnosis:** we collected blood samples from cases and contacts and sent the samples to the Viral Hemorrhagic Fever Reference Laboratory in Kenema District for testing. A rapid diagnostic test was also performed in the field to screen for Lassa fever. Additionally, RT-PCR and ELISA tests were conducted at the reference laboratory in Kenema District to confirm the diagnosis.

**Contact tracing:** a contact was any person who had direct or indirect contact with a case or surfaces contaminated by body secretions from a case in Kenema District from January 01 to March 31, 2019. We used a line-listing form developed by MoHS to list contacts.

**Environmental assessment:** the District Health management team assessed the case patients´ housing structure, waste location, collection, and disposal, and household hygiene and rodent infestation. We conducted ring trapping of rodents in each of the case patient´s residents and ten neighbor houses using Sharman traps and bait. The bait was a common food eaten by the residents, such as dry fish, groundnut, and rice. The trapping was done for two nights. After the trapping, we collected specimens for laboratory investigation. Rodent specimens were further classified by a reference laboratory at Tulane University, USA, to identify the species that cause the infection of the case patients.

**Consent and institutional review board approval:** ethical approval was not applicable to the investigation of these case-patients because it is part of a routine epidemic prone disease notification and case investigation. Verbal consent was granted by the case patients and their families to conduct the investigation and collect samples. No personal identifiable information was disclosed to other parties except to the investigators.

## Results

The investigation confirmed two children in the Kenema District with Lassa fever. The children lived in the Dodo chiefdom and Nongowa chiefdom, Kenema District. Both case-patients were notified by Healthcare workers (HCWs) working at Panguma Hospital and KGH.

### Demographic and clinical findings

**Case-patient one:** on February 10, 2019, an eight-year-old male developed fever (>39.5°C), and sore throat. On 18^th^ February 2019, the case patient was presented to the general outpatient department at Panguma Hospital after eight days of persistent fever (39.5°C), generalized body aches and pain, sore throat, and vomiting. On arrival at the hospital, the attending clinician screened the case-patient and diagnosed pneumonia and malaria, and recommended admission. The case patient was admitted to a hospital and treated for malaria and pneumonia. On 20^th^ February 2019, Lassa fever was suspected when the case patient began to bleed from their orifices. A blood sample was collected and confirmed that the case patient was infected with Lassa fever. An ambulance was dispatched to transfer the case patient to KGH, but the case patient died before the ambulance arrived.

**Record review and active case search:** the investigation revealed that no suspected cases were found in the general registers, case base forms, rumor logbook, and district database. Additionally, no suspected cases were found in the community where the case patients were living.

**Contact tracing:** eight contacts were listed for case-patient one, four HCWs, and four family members. Ten contacts were listed for case-patient two, four HCWs, and six family members. None of these contacts developed any symptoms compatible with Lassa fever 21 days with daily follow-up.

**Epidemiological findings:** the two case-patients had no reported contact with people who had recently been diagnosed with Lassa fever or had signs and symptoms of Lassa fever. The case patients did not travel outside their village.

**Environmental assessment:** the two case-patients lived in mud houses without paved floors. The households were in a wetland close to a garbage dumping site. Households were crowded and food was poorly stored. Rodent droppings and boreholes were observed in the homes of each case patient. The surrounding environment was untidy with rubbish scattered around their houses.

**Rodent trapping:** twenty-two traps were set using Sharman trap and bait. One trap was placed in each case patient´s residence and ten neighboring houses. Three rodents were caught, and one tested positive by RT-PCR for Lassa fever. We observed rodent droppings in both case patients´ homes while rodent boreholes were found only in the case-patient one´s residence.

## Discussion

Lassa fever was confirmed by symptoms and laboratory tests in two children living in Kenema District, Sierra Leone. The clinicians did not suspect Lassa fever when the case-patients presented to the healthcare facilities. Both case-patients were diagnosed and treated for malaria and pneumonia which are common in Kenema District and are part of the differential diagnosis of Lassa fever. Clinical diagnosis of Lassa fever is a challenge for clinicians as it is similar to other infections such as severe malaria, typhoid fever, and other viral hemorrhagic fevers [8]. Malaria is endemic in Kenema District, and clinicians usually empirically treat patients for malaria and suspect Lassa fever when patients develop bleeding from orifices, a typical symptom of Lassa fever, however, the prognosis of patients after developing bleeding is very poor. Late suspicion of Lassa fever contributed to the death of both case-patients from Lassa fever. The Lassa fever case fatality rate among the general population in Sierra Leone, specifically in Kenema District, is approximately 70%. The Lassa fever case fatality rate is about 20% in other developing countries and 20% among children [14,15]. Due to similar clinical presentation with other febrile illnesses, Lassa fever diagnosis remains a major challenge among health care workers in developing countries. Rodents in case-patients´ residences were positive for Lassa fever virus. Assessment of trapped rodents in this investigation indicated that the proportion of rodents captured with Lassa fever (33%) was higher than in a four-year study conducted in Upper Guinea (0.3%) [16]. Evidence of rodent boreholes and droppings suggested that the case-patients were infected by rodents. The two case-patients lived in conditions conducive to a rat infestation in an area endemic with Lassa fever, with rodents positive for Lassa fever living around their homes. Transmission of Lassa fever to close contacts usually only occurs while the patient has symptoms. However, an infected person can excrete Lassa fever in urine between 3 and 9 weeks after the onset of illness and via semen for up to 3 months. The investigation revealed that the two case-patients had no contact with a confirmed or probable case of Lassa fever or people with clinical signs and symptoms of Lassa fever and did not have a history of travel to a Lassa fever outbreak area three weeks before the onset of symptoms and no epidemiological link between the two case-patients. Thus, the infection was more likely to be attributed to a source at their residence.

## Conclusion

The investigation confirmed two children in the Kenema District contracted Lassa fever. No other cases were detected, and no contacts developed symptoms. Late detection of Lassa fever in these children may have contributed to their death. We concluded that the most likely source of infection was contact with rodents or droppings at the case-patients´ residence. The case-patients had no history of travel or contact with a person known to have been infected with Lassa fever. Rodents living near their residents were positive for Lassa fever. Improvements in environmental sanitation and hygiene by maintaining clean households and by appropriate food storage in leak-proof containers and disposing garbage far from houses may reduce opportunities for Lassa fever transmission. Public health measures included training HCWs on Lassa fever case detection, clinical case management and intensified community health education on Lassa fever disease. We conducted community education on environmental hygiene practices and on early health care seeking when Lassa fever symptoms are suspected.

### What is known about this topic

- *Lassa fever (LF) is a viral hemorrhagic epidemic prone disease that is endemic in West Africa including Sierra Leone;*
- *Lassa fever in West Africa accounts for an estimated 300,000 new cases and 5000 deaths every year*.

### What this study adds

- *It confirmed two children in the Kenema District contracted Lassa fever virus which finally led to their death;*
- *Late detection of Lassa fever in these children might have contributed to their death;*
- *The most likely source of infection was contact with rodents or droppings at the case-patients' residence.*
